# Historical ecology of riverine fish in Europe

**DOI:** 10.1007/s00027-015-0400-0

**Published:** 2015-07-07

**Authors:** Gertrud Haidvogl, Richard Hoffmann, Didier Pont, Mathias Jungwirth, Verena Winiwarter

**Affiliations:** Institute of Hydrobiology and Aquatic Ecosystem Management, University of Natural Resources and Life Sciences Vienna, Max Emanuelstraße 17, 1180 Vienna, Austria; Department of History, 2140 Vari Hall, York University, 4700 Keele St., Toronto, ON M3J 1P3 Canada; Irstea UR HBAN, 1 rue Pierre-Gilles de Gennes—CS 10030, 92761 Antony, France; Faculty of Interdisciplinary Studies, Institute of Social Ecology, Alpen-Adria-Universität Klagenfurt, Schottenfeldgasse 29, 1070 Vienna, Austria

**Keywords:** Historical ecology, History of fish, European rivers, Historical sources, Integrated river basin management

## Abstract

The temporal dynamic of riverine ecosystems and their fish communities and populations has been addressed in ecological theory and management for several decades. A growing number of case studies on the historic development especially of European and North American rivers have been published. Nonetheless, a theoretical debate about the contributions and limits of historical approaches and interdisciplinary co-operation is lacking. This article presents a brief overview of the role of history in river and fish ecology and suggests historical ecology as a scientific field that can offer a framework for future research. Based on case studies compiled in this special issue on the “Historical ecology of riverine fish in Europe”, we draw conclusions on long-term changes of fish communities, on fisheries, aquatic ecosystem management and past habitat alterations and the potential of archaeological remains and written sources to study them. We discuss how modelling of historical fish data can help elucidate the effects of climate change and human influences on rivers and fish. Finally, we account for the necessity to consider appropriate spatial and temporal scales. In conclusion we call for future comparative studies on continental and global scales and methodological development, which can benefit especially from recent advances in marine historical ecology. We suggest that future interdisciplinary studies of ecologists, hydrologists, historians and archaeologists can reveal the history of riverine ecosystems as socio-ecological systems, addressing both their natural dynamics and human dimension. Such an endeavor can also support developing management plans for habitat restoration and conservation against the background of global change.

## The temporal dimension and history in river and fish ecology

The dynamic nature of ecosystems has been addressed for several decades. Disturbance theory is often identified as an important milestone of the debate. White and Pickett (1985) defined disturbance as a “relatively discrete event in time that disrupts ecosystem, community or population structure and changes resources, substrate availability, or the physical environment”. Resh et al. (1988) applied the idea of disturbance regimes in river ecology. In 1989, Ward conceptualized lotic ecosystems as 4-dimensional (Ward 1989). He emphasized the role of temporal dynamics apart from the three spatial dimensions, i.e. the longitudinal, the lateral and the vertical and pointed to the difficulties to distinguish low-level anthropogenic perturbations from normal variations resulting from long-term natural cycles (Ward 1989; see also Hohensinner et al. 2011). Later, concepts rooted in the idea of an ecological equilibrium have been challenged, taking into account species dispersal and metapopulation theory (Hanski 1999), the role of spatial heterogeneity on ecological processes and the importance of fluxes between ecosystems (Pickett and Cadenasso 1995). Ecosystems are now seen as unstable, open, hierarchical and scaled (O‘Neill 2001).

Without referring explicitly to the progress in ecological theory, a growing number of case studies on the historical development of riverine ecosystems have been published since the end of the 1980s. For European river systems, Petts et al. (1989) presented long-term changes of river morphology and hydrology. They also accounted for biological studies with a focus on fish. Similar publications followed for the Americas, even treating fish specifically (see e.g. Rinne et al. 2005). On a general level, Downs and Gregory (2004) identified six historical periods of river use and according management practices. They distinguish hydraulic civilizations, preindustrial societies, the period of the industrial revolution, the late 19th to mid-20th century, the second half of the 20th century and finally, the period since the late 20th century, which accounts for integrated river basin management, re-regulation of flow, mitigation, enhancement and restoration techniques as well as hybrid and bioengineered revetments. In terms of biodiversity change, three periods are usually differentiated with reference in particular to the so-called “Columbian exchange”. Notwithstanding earlier species‘transfers on a smaller scale, the “discovery” of the Americas by Europeans in 1492 is perceived as a turning point in the history of ecosystems and their biota (see especially Crosby 1972, 1986). In the second half of the 19th century, the industrial revolution with its improved means of transport triggered large-scale and frequent exchange of species. Besides deliberate introductions, for which the rainbow trout can be cited as an example for some European freshwater systems, this led also to the translocation of species to new habitats where they were considered as non-native or even invasive (di Castri 1989 as an early study).

In the 1990s, historical approaches gained importance in ecosystem management. Restoration ecology was increasingly relying on historically based reference conditions and “historical ecology” has been adopted as a specific approach to study past ecosystems and their development (see e.g. Swetnam et al. 1999; Egan and Howell 2001). Freshwater system management was no exception. When the European Water Framework Directive (WFD, EC 2000) came into force, the idea of natural, i.e. anthropogenically undisturbed aquatic systems as a target of restoration even received legal status. Although the WFD is not interested in historical conditions as such, it stimulated related research, as it requires defining a high ecological status, reflecting conditions in the absence of human influence (e.g. Jungwirth et al. 2002). Recent studies in European fish ecology thus often focus on the historical development of fish species and fish communities. Long-term research in fish ecology, however, still deals mainly with the last two centuries.

In recent years, several scientists have scrutinized the concept of historically based reference conditions. Such an approach implicitly assumes a stable state of ecosystems, thus ignoring the advancements in theoretical ecology. Furthermore, fundamental changes of ecosystems, regardless whether humankind or nature triggered them, usually prevent return to previous conditions (Bouleau and Pont 2015). The difficulties of reconstructing past ecosystems including species composition, abundance or biomass based on the available historical sources could be a further shortcoming. But this does not reduce the interest in historical contributions to ecology. In contrast, recent publications emphasize a clearer and more mature role of history. Bearing in mind natural dynamics and the long-lasting influences of societies on ecosystems, river and fish ecologists suggest investigating the long-term development of riverine landscapes as co-evolution of ecological and climatic conditions on the one hand and human influences on the other (e.g. Dufour and Piegay 2009; Humphries and Winemiller 2009; Pont et al. 2009; Labay et al. 2011). A restoration ecology acknowledging that humankind has entered the “Anthropocene” will face new challenges triggered by future climate change and the resulting novel ecosystems and ‘no-analogue’ communities (Hobbs 2012; Mascaro et al. 2013; Ellis 2015). Against this background, Higgs et al. (2014) identified nine different types of historical knowledge, many of which will advance existing concepts and principles of restoration ecology. In such approaches, history serves as a guide for determining restoration goals (see e.g. Suding et al. 2015).

## Historical ecology of rivers and fish

Historical studies in river and fish ecology can greatly benefit from the existing scientific framework and concepts of historical ecology—according to Rick and Lockwood (2013) “… the use of historic and prehistoric data (e.g. paleo-biological, archeological, historical) to understand ancient and modern ecosystems, often with the goal of providing context for contemporary conservation. A fundamental goal of historical ecology is to understand past and present human-environment interactions, but it is also concerned with understanding natural variation before and after human arrival”. The term “Historical Ecology” has been used since the middle of the 20th century for a variety of studies of past human-nature relationships. The scope of the field is wide, as studies have been produced in at least four different scientific disciplines, i.e. in history, ecology, geography and anthropology (see esp. Szabo 2014 for a recent review of historical ecology). It is beyond the objective of this article to deal in detail with the complex commonalities and differences between the various approaches. We would, however, like to emphasize that historical ecology studies focus mainly on past ecological conditions and their implications for present and future ecosystem management.

Despite their potentially large role in ecology and biodiversity research (see e.g. Verheyen et al. 2004; Rhemtulla and Mladenoff 2007; Wiens et al. 2013), historical studies of river and freshwater fish ecology are rarely discussed as an independent, interdisciplinary endeavor requiring ecological as well as historical knowledge. But historical ecology as a research field can contribute to the study the legacies of previous human interventions into riverine landscapes and the consequences of past anthropogenic and other disturbances, which influence the present and future development. Resulting knowledge can support management plans for future habitat restoration, conservation, and adaptation to global change.

Historical ecology is more than doing ecological studies using monitoring data from the last few decades. A long-term view is indispensable, although data on historical biodiversity are particularly difficult to obtain (see e.g. Beck 2013). Studies of past riverine fish communities and populations are faced with incomplete data especially when it comes to abundance and biomass and often even with regard to species presence. Before field monitoring with standardized and reproducible sampling protocols became more common in the second half of the 20th century, fish biological surveys often relied only on interviews with fishermen. Early exceptions stem from surveys of hydropower dams and their impacts on fish migration (e.g. Steinmann et al. 1937). The study of Pont et al. 2015 (this issue) is based on another exception, as for the Rhone catchment field surveys were already being done in the 1930s (see also Carrel 2002). Mostly, sources for studies of past fish communities and populations were produced in the context of commercial and artisanal fishery, fish trading and fish consumption (see Haidvogl et al. 2014 for an overview). They do not enable direct comparison with present field sampling, so specific methods have to be developed to identify changes.

Apart from the specific types, limits and possibilities of sources, historical ecology holds that humans are more than a mere impact on ecological integrity. Humans and the way they used and exploited aquatic ecosystems and their resources have to be studied as an important feature of past river systems and fish communities in their own right. Although the societal imprint on rivers increased exponentially since the onset of industrialization, due to new technological means and the possibilities of using fossil fuels, humans have influenced river systems and fish for centuries, if not millennia. Fishing and overexploitation caused species extinction before 1700 (see Kottelat and Freyhof 2007). Mill weirs or large-scale land-use change modified habitats and subsequently fish (e.g. Hoffmann 1996; Walter and Merrits 2008; Giosan et al. 2012).

The investigation of ecological and human systems as intertwined or hybrid is usually the domain of environmental history. Environmental history has contributed a wealth of studies on the long-term development of aquatic systems, for instance as a source of food, energy and waste/wastewater discharge but also as a threat due to floods or droughts. For fish populations of medieval European rivers, several publications of Hoffmann (e.g. Hoffmann 1987, 1994, 1996, 2005, 2008; Hoffmann and Sonnlechner 2011) have laid the groundwork. For the modern period, studies of freshwater systems and their fish often concentrate on North American rivers or focus on diadromous species (e.g. Steinberg 1991; Bogue 2000; Evenden 2004; Coates 2006; Henshaw 2011). This is in sharp contrast to marine environments and their fish communities where the HMAP project (History of Marine Animal Populations) as a large research endeavor has contributed impressive results (see for instance Lotze et al. 2014; Lotze and McClenachan 2014).

Despite the fact that sometimes the two terms ‘historical ecology’ and ‘environmental history’ are used as synonyms (see e.g. Szabo 2014; Winiwarter 2004), we suggest to distinguish between these two (inter-)disciplines. Historical ecology of riverine fishes focuses on the aquatic environment as an ecosystem, i.e. on the reconstruction of fish communities and populations in conjunction with past habitat conditions including climate change. Just like environmental history, this requires the interdisciplinary cooperation of ecologists, hydrologists, historians and archaeologists, but in addition a specific treatment and interpretation of available sources and methods is necessary (see e.g. Haidvogl et al. 2014).

Archaeology, finally yet importantly, contributes valuable results for understanding past fish ecological conditions (see for instance Van Neer 1994). Modern methods of genetics and isotope analysis enhanced the potential of investigating fish remains and help, among other contributions, to overcome the problem of identifying the provenance of excavated fish remains (Ludwig et al. 2002; Orton et al. 2011; Chassaing et al. 2013).

## An overview of the case studies of this special issue

### Spatial and temporal coverage and representativeness of the case studies

**Fig. 1:**
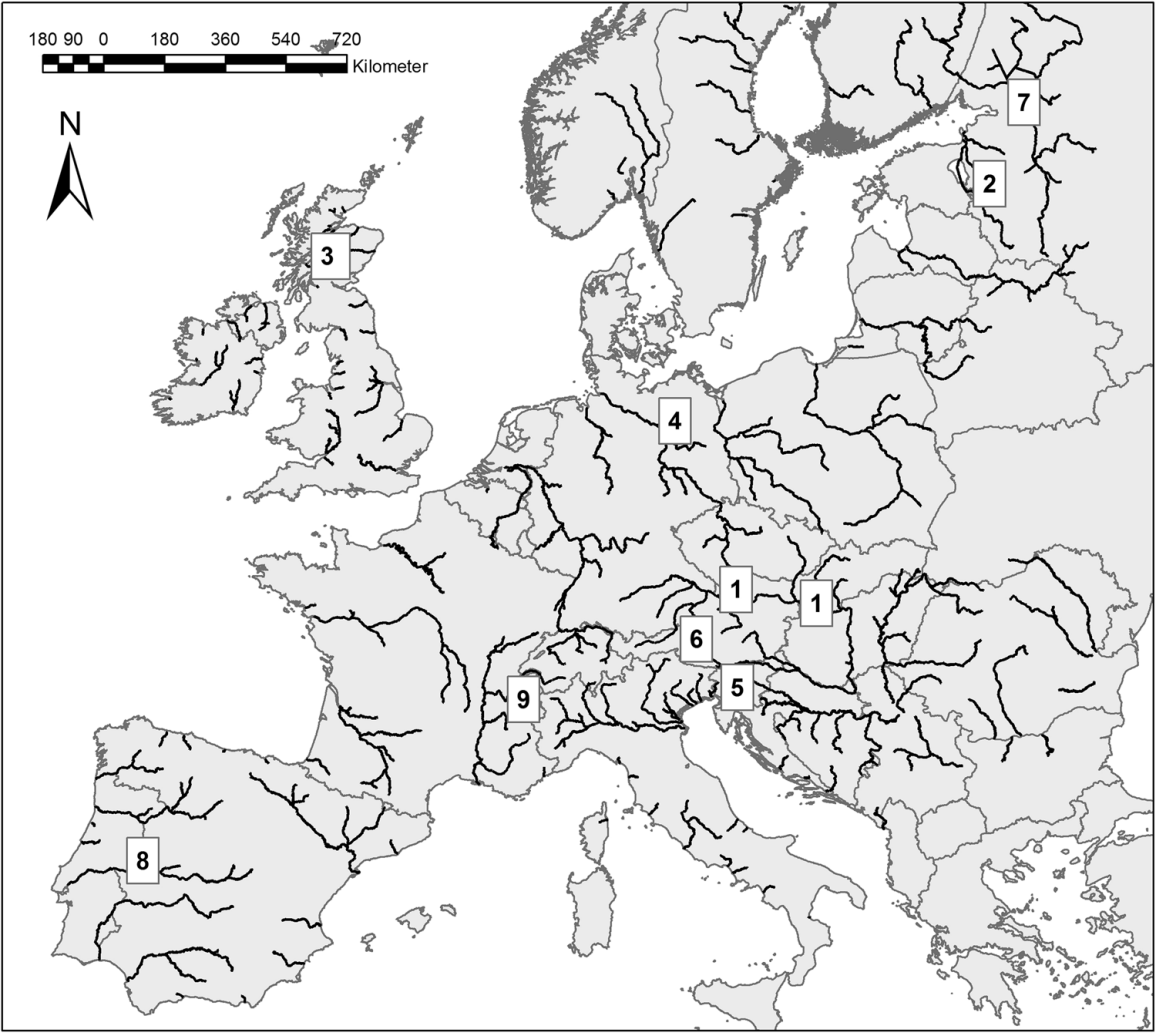
The locations of the rivers presented in this special issue. Only rivers with a Strahler stream order >5 are shown. *1* Austrian and Hungarian Danube, *2* Velikaya and Lake Peipus, *3* Rivers of the Scottish North, *4* Elbe, *5* Irdijca, *6* Salzach, *7* Neva and Gulf of Finland, *8* Tagus, *9* Rhone

The papers of this special issue present and discuss fish species and fish community changes in a variety of rivers and connected lakes and marine environments. They consider fluvial systems from various European biogeographical regions, comprising Baltic and Eastern continental rivers (Neva and Gulf of Finland, Velikaja and Lake Peipus), British rivers (eastern Scottish tributaries of the North Sea), rivers of the central highlands and plains (upstream section of Elbe; Saône catchment), continental and Atlantic rivers (Lower section of Elbe), alpine and mountainous rivers of central and southern Europe (Salzach, Idrijca; Rhone tributaries), Iberian rivers (Tagus), and sections of the Danube (see Fig. 1). Fish species diversity of the investigated rivers differs, as European aquatic systems show a clear west-east and north south gradient, where the Danube has the highest species richness and (together with south European rivers) many endemic species (Reyjol et al. 2007).

The following contributions deal with single species or whole fish communities. Hoffmann (2015) and Wolter (2015) (both this issue) focus on Atlantic salmon (*Salmo salar*) in northern and central European rivers. Segurado et al. 2015 (this issue) investigate allis shad (*Alosa alosa*) and sea lamprey (*Petromyzon marinus*) in the Portuguese Tagus basin. Torkar and Zwitter (2015) concentrate on trout and a few other resident freshwater species, which are part of the Idrijca fish community.

Most studies, however, address a multitude of species, such as Pont et al. 2015 (this issue) or focus on entire fish communities as far as this is possible on the basis of historical written sources and archaeological remains (Galik et al. 2015; Yurtseva et al. 2015; Haidvogl et al. 2015; Lajus et al. 2015). Lajus et al. (2015) take into account some marine fish species in order to contextualize shifts in fishing pressure from the Neva River towards the Baltic Sea.

**Fig. 2:**
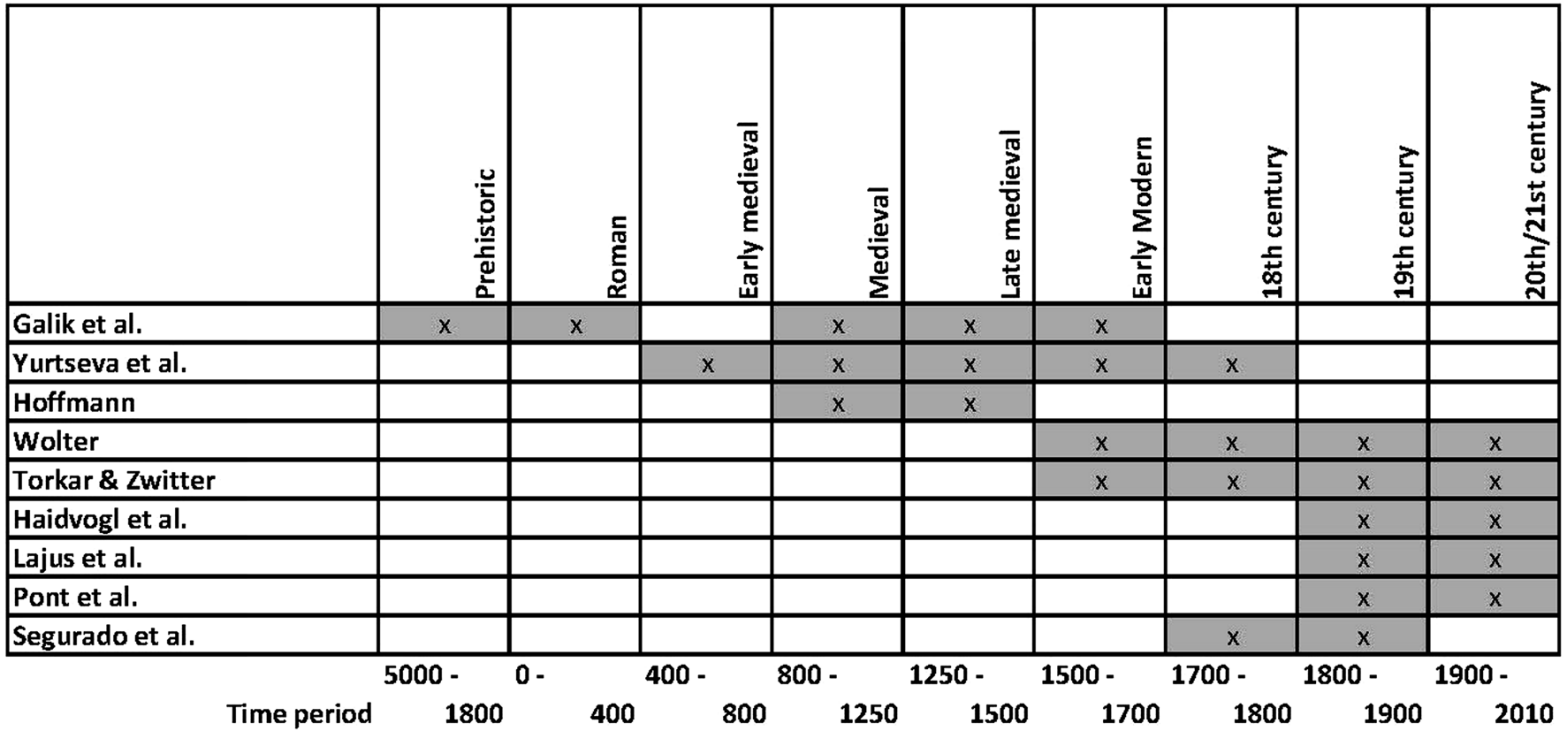
Temporal coverage of the case studies presented in this special issue

The studies in this issue focus on different periods from pre-history and Roman times up to the 20th century and consider predictions of future biodiversity against the background of climate change (see Fig. 2). For Austrian Danube tributaries, some of the archaeological fish remains stem from excavations of Neolithic, Copper and Bronze Age but most archaeological remains discussed in Galik et al. (2015) and Yurtseva et al. (2015) date from Roman, medieval and early modern times. Hoffmann 2015 (this issue) addresses the high and later middle ages, using primarily written sources. The majority of the papers deal with the early modern and modern period; there is a clear dominance of data from the 19th and 20th century.

The case studies use archaeological fish remains or written documents, including for the latter both archival sources and printed documents. All types of historical sources have their potential and limitations. For community studies, archaeological remains require specific sieving techniques that allow recovering bones of small species. In addition, taphonomy has to be considered, i.e. species-specific temporal differences in the decay of bones depending on their fat content (Szpak 2011). The availability of written documents changed considerably over the centuries. Only a few documents are available from Roman times, but relevant records were compiled in some instances since the High Middle Ages, roughly from the 13th century onwards. Fish-ecological surveys are a rather recent effort, starting in the 2nd half of the 20th century. In contrast to most other aquatic biota, studies of past fish communities and populations can often utilize fishery catch data. Before the middle of the 20th century, these are certainly the most promising sources. However, such records—if prepared at all—have often been lost. In addition, they focus only on species of commercial interest. The typical shortcomings of historical catch data is their incompleteness in terms of fish communities, the lack of abundance information and the lack of longer time series. Many other source types from fisheries, trade and consumption exist and have been explored in the different case studies.

### Topics addressed and trends identified

#### Millennial-scale changes and the potential of archaeological remains

Archaeological remains allow investigating long time spans and changes on larger spatial scales. They originate from excavations of castles, monasteries, or historic settlements where e.g. taverns or waste deposits were investigated. The number of species found depends on the recovery or sampling technique employed; careful sieving commonly yields the best results (see Galik et al. 2015). Knowledge about the local origin of species depends on the possibilities of trading fish. As Galik et al. (2015) demonstrate, marine fish appear in excavations along the Austrian Danube already in Roman times. This clearly shows that fish consumed did not only originate from local waters. The Danube sections investigated revealed different species compositions reflecting distinct habitat conditions. Comparing archaeological remains and written documents in the Austrian and the Hungarian Danube from overlapping periods, i.e. the 16th century, showed that in the fish remains some species were missing. In excavations along the lower Velikaya River and Lake Peipus not only fish remains but also fishing gear was found. Together with the predominant identified fish species, these artefacts demonstrated that fishing activities had shifted from the river towards the lake. Decreasing average lengths especially of bream and perch as well as a shift of dominant species towards smaller ones can be interpreted as signs of fishing pressure and overexploitation. In the Velikaya River and Lake Peipus the decrease in lengths was, however, moderate and has to be interpreted in comparison with data from the 20th century. For the Austrian Danube and some tributaries, the decrease was much more pronounced, in particular for carp. For the latter, this can be attributed to effects of artificially bred individuals from fish farms and/or a standardization of fishing gear in accordance with newly established fishing laws for the Late Middle Ages and later periods.

#### Fisheries, aquatic ecosystem management and habitat alteration

It is often assumed that throughout the centuries and especially over the last 200 years, fish stock decline was and is a continuous trend. Our case studies show that at least for some species this development might be true on a large scale, but local situations were of considerable variety. As in marine environments, fishing pressure was surely one of the earliest causes of fish community changes in river systems. Quantitative data are lacking, but the imposition of fishing regulations since the late Middle Ages indicates a decline in specific species due to overexploitation. Historical research recognizes in such laws an attempt by political authorities to assert power and control, which has to be figured into the assessment of regulations as sources for the identification of overexploitation (e.g. Sonnlechner and Winiwarter 1999; Warde 2006; Hoffmann and Sonnlechner 2011). Further, in river systems the effects of fishing are overlain by and interact with other human influences and climate change. Thus, it is rarely possible to identify the contribution of a single stressor.

In terms of fishery management, Hoffmann 2015 (this issue) demonstrated that salmon, a highly prized commodity, remained abundant at least until the early 16th century in Scottish rivers while in other European regions such as around the North Sea the species was already in decline due to overexploitation. The Scottish Highlands were an area of cattle farming rather than of arable land for crops. Therefore, erosion and subsequent input of sediment and hydrological alteration of streams were minimized, providing better conditions for salmon.

Intense mining of mercury ore started in Idrija (Slovenia) in the late 15th century. The site was the second largest mercury mine in the world until its closing in the 1990s. It caused severe pollution of the Idrijca river system. Direct effects on fish were a consequence of mercury-contaminated water (and soils) especially in periods of high intensity of ore processing but also during and after large fires in the mines, when large quantities of water were released from the mines into the rivers. In addition, intensive fishing due to a growing human population in the Idrijca valley contributed to the exploitation of fish stocks. Indirect effects are likely a consequence of firewood and timber driving which took place in this area throughout the year. Astonishingly, at the end of the 19th century sections and tributaries of the Idrijca more distant from the mines were still reported as among the very productive river sections of Carniola. In the late 19th century, non-native brown trout (*Salmo trutta*) stocking started. It caused the decline of the native marble trout (*Salmo marmorata*). Dam construction in the 1930s resulted in the decline of catadromous eel.

In the Idrijca basin, log driving was regularly reported as a severe pressure on fish before the early 20th century. Firewood and larger timber were transported throughout the year including the spawning season of marble trout in November and December. This type of human river use has been studied with different results for the Salzach catchment. Here especially firewood for large-scale salt processing downstream was transported on the tributaries during a limited annual season, i.e. after the snow melt when higher discharges could be used. A statistical test using systematically recorded data on species presence and abundance classes from the turn of the 19th to the 20th century showed no significant difference between rivers with and without log driving. Apart from shortcomings of the available historical data and possible other influences on fish, specific forest and log driving management are a possible explanation of this result (Haidvogl et al. 2015).

It remains difficult to quantify the effects of human-induced habitat alteration before the 19th century e.g. due to land-use changes, shipping, bank protection, pollution and later also flood protection and their effects on fish. For the Elbe River, salmon catch data from the middle of the 16th to the middle of the 19th century were analyzed (Wolter 2015). Reports from the 18th century refer to a decline but do not quantify it. Only from the 19th century have more data been passed on. The catchment-wide study indicates that for the Elbe existing assumptions on historical spawning stocks of salmons have to be corrected from about 4000 individuals to 8000–10,000 individuals (Wolter 2015).

In the Neva and in the Gulf of Finland, changes in fishing sites, fishing gear and techniques are clearly visible in the 19 and 20th century. Until the 19th century, fishing in the lower Neva River was done mainly with weirs and nets. Sturgeon (*Acipenser sturio*), salmon and whitefish (*Coregonus lavaretus*) were the target species. By the end of the 19th century, fishing had shifted to coastal areas and focused on smelt (*Osmerus eperlanus*) and herring (*Clupea harengus*). Catch data show decreasing harvests of diadromous fish, which can be attributed most likely to a change of water transparency. The end of the 20th century is characterized by improved fishing technology. Large pelagic trawlers target herring offshore (Lajus et al. 2015).

#### Modelling, shifting baselines and climate change effects on fish

The effects of past climate change on fish were blended with those of fishing and human alterations of aquatic ecosystems. This makes it very difficult to identify the role of climate change for fish population changes. The effects of climate change in the past can nevertheless be studied as a single driver using species distribution models and historical climate data. Because water temperature was rarely measured before the 20th century, air temperature and precipitation data have been used by Pont et al. 2015 (this issue) as proxies to investigate changes in species occurrence at catchment level. The study discusses also possible effects of future climate change on fish. Integrating historical and future data offers the opportunity to test shifting baselines according to Pauly (1995) and to establish, whether the variability of a single species is within or outside of historical ranges. As shown in this issue for the Salzach and Rhône catchments, several fish species will in the future exhibit shifts in their distribution beyond historically observed values. This is in particular true for trout (*Salmo trutta*) or barbel (*Barbus barbus*) which are very sensitive to temperature (Pont et al. 2015).

For allis shad and sea lamprey, Segurado et al. (2015) analyzed changes of the functional connectivity in the Tagus basin during the 20th century. Employing a spatial graph approach, they demonstrated the effects of individual dams and identified the most destructive ones.

#### Spatial and temporal scale of investigations

The investigation of salmon catches from the Elbe catchment demonstrated the value and necessity of investigations on a larger spatial scale. As Wolter (2015) has been able to show, looking only at the variability of salmon catches at a specific site might be misleading. Discharge patterns matter, especially flood events that enabled salmon to migrate further upstream. This resulted in higher catches at salmon weirs in upstream sections of the Elbe and tributaries, respectively. Fishermen upstream benefitted also from the negative effects of channelization on the use of fishing gear (seine nets) in the lower Elbe. Their use was no longer possible because the large gravel bars that are required for seine nets had disappeared.

The importance of looking at longer periods was highlighted by the study of Pont et al. 2015 (this issue) on the effects of past climate change on the Salzach and Rhône catchments. Using species distribution models and historical air temperature data, they demonstrated that past climate change resulted in clear shifts of species occurrence already in the 19th century.

## Conclusions and recommendations for future research

The case studies of this special issue address different European rivers and their fish populations and communities. They present new approaches and methods of investigating historical data in an ecological context. They confirm existing knowledge but they also contribute to a broader and more complex picture of the issue. Fish as an important source of protein-rich food have been altered long before the industrial period. The various studies are place-based but collectively they certainly reflect developments in larger areas, which have undergone similar environmental change and societal conditions. Future comparative studies on a European scale might reveal commonalities but also differences in the co-evolution of rivers and societies.

In addition to contributing historical reference data for native species and a comprehensive understanding of long-term human-river-interactions, the investigation of non-native and invasive species dispersal will be an important future subject for a historical ecology of riverine fish. Kottelat and Freyhoff (2007) assert for Europe that on a catchment scale, about 40 % of native fish species disappeared due to fishing and habitat alteration. Simultaneously, the number of non-native species due to deliberate human action or as an unintended side-effect of trading and new artificial shipping channels increased. A total of 76 non-native species have been introduced in Europe and it has been estimated that 29 have established self-reproducing populations (Peter 2006 in Tockner et al. 2009).

The potential of historical sources is certainly not fully explored. Studies of riverine fish can benefit from the recent advancement in marine historical ecology, which integrates also studies of DNA isolated from ancient specimen (so-called aDNA) and stable isotopes (Lotze et al. 2014; Lotze and McClenachan 2014; Orton et al. 2011 to name just a few examples). In addition, marine historical ecology has come up with valuable concepts on the development of fish populations over time (e.g. “shifting baselines” or “fishing down marine food webs”, Pauly 1995; Pauly et al. 1998; Pitcher et al. 2001).

For thousands of years, humans tended to settle near rivers. The vast majority of major settlements are situated close to freshwater. Using rivers for transportation, energy provision, as ecosystem to exploit for food and as waste disposal contributed substantially to human livelihoods and wealth. Yet, floodplain settlements were also under threat of inundation. Humans have engineered flowing waters small and large to optimize the functions and eliminate or at least lower their risks. Rivers have been used as a wild habitat for protein harvesting for millennia. In their recent overview of sedimentation flux changes in the Anthropocene, Syvitski and Kettner (2011) present evidence that humans have started to engineer sediment flux 3000 years ago. They mention delta subsidence and the massive changes in coastal ecosystems due to sediment flow alteration, and assess that human influence is as important as climatic shifts such as the transition from the Pleistocene to the Holocene. Building weirs and water mills, drifting logs and changing sediment regimes via changes in land-use are among the most important of human influences. While these changes have been studied, seldom has the effect on fishes been in the focus and very few systematic studies exist to date. The pressures on freshwater habitat from overfishing, flood protection, regulation for transport and damming for energy, as well as from building artificial channels for mills combine differently in each river, and all rivers are very diverse when it comes to geology, morphology and substrate, seasonal dynamic, and other characteristics. As many river engineering projects were considered strategically important or of political interest as demonstrations of technological superiority or prestige in general, a multitude of natural and social factors has to be weighed in any specific case. The case studies in this volume show, how much can be gained from detailed studies. It is yet too early to ask, if from those cases, new patterns will become visible. One would need more comparative studies for that. A comparison with Asian rivers (Dudgeon 2011) might be an avenue to pursue.

We agree with Higgs et al. (2014) that the future role of history in (restoration) ecology should be more comprehensive than focusing on reference conditions. Especially in the European setting, long-term changes of various origins and millennia of human use mean “pristine” aquatic systems could only predate the late Pleistocene and river ecologies may have changed several times since the retreat of the glaciers. Any baseline is an arbitrary human choice. For the future, it is evident that against the background of global change, habitat conditions and subsequently fish species’ distribution will change, regardless of human influences, which might exacerbate or mitigate the effects of such changes. The terms “novel ecosystems” and “no-analogue communities” have been coined to address such trends in the past and in the future (Hobbs et al. 2013; Williams and Jackson 2007).

Humans have intervened directly by catching and stocking fish, but their indirect interventions have been just as important for fish distribution and abundance. This volume pioneers the interdisciplinary effort of historical ecology, which is necessary to elucidate how fishes were affected, when, where and why.

## References

[CR1] Beck C (2013). Histoire et Biodiversité: un (im)possible marriage? Revue du Nord, Hors série. Collection Art et Archéologie.

[CR2] Bogue MB (2000) Fishing the Great Lakes. An environmental history, 1783–1933. University of Wisconsin Press, Madison

[CR3] Bouleau G, Pont D (2015). Did you say reference conditions? Ecological and socio-economic perspectives on the European Water Framework Directive. Environ Sci Policy.

[CR4] Carrel G (2002). Prospecting for historical fish data from the Rhone River basin: a contribution to the assessment of reference conditions. Archiv fuer Hydrobiologie.

[CR5] Chassaing O, Desse-Berset N, Duffraisse M, Hughes S, Hänni C, Berrebi P (2013). Palaeogenetics of western French sturgeons spotlights the relationships between Acipenser sturio and Acipenser oxyrinchus. J Biogeogr.

[CR6] Coates P (2006). Salmon.

[CR7] Crosby A (1972) The Columbian exchange. Biological and cultural consequences of 1492. Greenwood Press, Westport

[CR8] Crosby A (1986) Ecological imperialism. The biological expansion of Europe, 900–1900. Cambridge University Press, Cambridge

[CR9] diCastri F, Drake JA, Mooney HA, di Castri F, Groves RH, Kruger FJ, Rejmanek M, Williamson M (1989). History of biological invasions with special emphasis on the old world. Biological Invasions: a global perspective.

[CR10] Downs PW, Gregory KJ (2004). River channel management: towards sustainable catchment hydrosystems.

[CR11] Dudgeon D (2011). Asian river fishes in the Anthropocene: threats and conservation challenges in an era of rapid environmental change. J Fish Biol.

[CR12] Dufour S, Piégay H (2009). From the myth of a lost paradise to targeted river restoration: forget natural references and focus on human benefits. River Res Appl.

[CR13] Egan D, Howell EA (2001) The historical ecology handbook: a restorationist’s guide to reference ecosystems. Island Press, Washington. D C

[CR14] Ellis E (2015). Ecology in an anthropogenic biosphere. Ecol Monogr (online first).

[CR15] European Commission (2000) Directive 2000/60/EC of the European parliament and of the council establishing a framework for the community action in the field of water policy (EU-Water Framework Directive). http://eur-lex.europa.eu/LexUriServ/LexUriServ.do?uri=CELEX:32000L0060:EN:NOT. Accessed 2 June 2015

[CR16] Evenden M (2004). Fish versus power. An environmental history of the Fraser River.

[CR17] Galik A, Haidvogl G, Bartosiewicz L, Guti G, Jungwirth M (2015) Fish remains as a source to reconstruct long-term changes of fish communities in the Austrian and Hungarian Danube. Aquat Sci (this issue) 10.1007/s00027-015-0393-8 PMC4525806 26257501

[CR18] Giosan L, Coolen MJL, Kaplan JO, Constantinescu S, Filip F, Filipova-Marinova M, Kettner AJ, Thom N (2012). Early anthropogenic transformation of the danube-black sea system. Sci Rep.

[CR19] Haidvogl G, Lajus D, Pont D, Schmid M, Jungwirth M, Lajus J (2014). Typology of historical sources and the reconstruction of long-term historical changes of riverine fish: a case study of the Austrian Danube and northern Russian rivers. Ecol Freshw Fish.

[CR20] Haidvogl G, Pont D, Dolak H, Hohensinner S (2015) Long-term evolution of fish communities in European mountainous rives: past log-driving effects, river management and species introduction (Salzach River, Danube). Aquat Sci (this issue) 10.1007/s00027-015-0398-3 PMC4550260 26321854

[CR21] Hanski I (1999). Metapopulation ecology.

[CR22] Henshaw RE (2011) An environmental history of the Hudson River. Human uses that changed the ecology, ecology that changed human uses. State Univeristy of New York Press, Albany

[CR23] Higgs E, Falk DA, Guerrini A, Hall M, Harris J, Hobbs RJ, Jackson ST, Rhemtulla JM, Throop W (2014). The changing role of history in restoration ecology. Front Ecol Environ.

[CR24] Hobbs R (2012) Environmental management and restoration in a changing climate. In: van Andel J, Aronson J (eds) Restoration ecology. The new frontier. 2nd Edn. Wiley-Blackwell, Chichester

[CR25] Hobbs R, Higgs E, Hall C (2013). Novel Ecosystems.

[CR26] Hoffmann R (1987) The protohistory of pike in western culture. In: Crossman EJ, Casselman J (eds) An annotated bibliography of the Pike Esox lucius. Life Sciences Miscellaneous Publications. Royal Ontario Museum, Toronto, pp vii–xvi

[CR27] Hoffmann R (1994) Remains and Verbal Evidence of Carp (Cyprinus carpio) in Medieval Europe. In: Van Neer W (ed) Fish exploitation in the past. Proceedings of the 7th Meeting of the I.C.A.Z. Fish Remains Working Group, Annales du Musée Royal de l’Afrique Central, Sciences Zoologiques 274:139–150

[CR28] Hoffmann R (1996). Economic development and aquatic ecosystems in medieval Europe. Am Hist Rev.

[CR29] Hoffmann R (2005) A brief history of aquatic resource use in medieval Europe. In: Lotze HK, Reise K (eds) Helgoland marine research (special issue Ecological history of the Wadden Sea) 59(1): 22–30

[CR30] Hoffmann R, Herrmann B (2008). Medieval Europeans and their Aquatic Ecosystems. Beiträge zum Göttinger Umwelthistorischen Kolloquium 2007-2008, Graduiertenkolleg Interdisziplinäre Umweltgeschichte.

[CR31] Hoffmann R (2015) Salmo salar in late medieval Scotland: competition and conservation for a riverine resource. Aquat Sci (this issue)

[CR32] Hoffmann R, Sonnlechner Ch (2011) Vom Archivobjekt zum Umweltschutz: Maximilians Patent von 1506. Studien zur Wiener Geschichte. Jahrbuch des Vereins für Geschichte der Stadt Wien 62/63:79–133

[CR33] Hohensinner S, Jungwirth M, Muhar S, Schmutz S (2011). Spatio-temporal habitat dynamics in a changing Danube River landscape 1812–2006. River Res Appl.

[CR34] Humphries P, Winemiller K (2009). Historical impacts on river fauna, shifting baselines, and challenges for restoration. Bioscience.

[CR35] Jungwirth M, Muhar S, Schmutz S (2002). Re-establishing and assessing ecological integrity in riverine landscapes. Freshw Biol.

[CR36] Kottelat M, Freyhof J (2007). Handbook of European freshwater fishes.

[CR37] Labay B, Cohen AE, Sissel B, Hendrickson DA, Martin FD, Sarkar S (2011). Assessing historical fish community composition using surveys, historical collection data, and species distribution models. PLoS One.

[CR38] Lajus D, Glazkova J, Sendek D, Khaitov V, Lajus J (2015) Dynamics of fish catches in the eastern Gulf of Finland (Baltic Sea) and downstream of the Neva River during the 20th century. Aquat Sci (this issue)

[CR39] Lotze HK, McClenachan L, Bertness MD, Silliman BR, Bruno JF, Stachowicz JJ (2014). Marine Historical Ecology: informing the future by learning from the past. Marine community ecology and conservation.

[CR40] Lotze H, Hoffmann R, Erlandson J (2014) Lessons from Historical Ecology and Management. In: Fogarty M, McCarthy J (eds) Marine ecosystem-based management. The sea—ideas and observations on progress in the study of the seas, Vol 16. Harvard University Press, Cambridge Massachusetts and London England, pp 17–55

[CR41] Ludwig A, Debus L, Lieckfeldt D, Wirgin I, Benecke N, Jenneckens I, Williot P, Waldman JR, Pitra C (2002). Fish populations: when the American sea sturgeon swam east. Nature.

[CR42] Mascaro J, Harris JA, Lach L, Thompson A, Perring MP, Richardson DM, Ellis E, Hobbs RJ, Higgs ES, Hall CM (2013). Origins of the novel ecosystems concept. Novel ecosystems: intervening in the new ecological world order.

[CR43] O‘Neill RV (2001). Is it time to bury the ecosystem concept? (with full military honors, of course!). Ecology.

[CR44] Orton DC, Makowiecki D, deRoo T, Johnstone C, Harland J, Jonsson L, Heinrich D, Bødker Enghoff I, Lougas L, Van Neer W, Ervynck A, Hufthammer AK, Amundsen C, Jones AKG, Locker A, Hamilton-Dyer S, Pope P, MacKenzie BR, Richards M, O’Connell TC, Barrett JH (2011). Stable isotope evidence for late Medieval (14th–15th C) Origins of the Eastern Baltic Cod (Gadus morhua) fishery. Plos One.

[CR45] Pauly D (1995). Anecdotes and the shifting baseline syndrome of fisheries. Trends Ecol Evol.

[CR46] Pauly D, Christensen V, Dalsgaard J, Froese R, Torres FJ (1998). Fishing Down Marine Food Webs. Science.

[CR47] Petts GE, Moller H, Roux AL (1989). Historical change of large alluvial rivers: Western Europe.

[CR48] Pickett STA, Cadenasso ML (1995). Landscape ecology: spatial heterogeneity in ecological systems. Science.

[CR49] Pitcher TJ (2001). Fisheries managed to rebuild ecosystems? Reconstructing the past to salvage the future. Ecol Appl.

[CR50] Pont D, Piégay H, Farinetti A, Allain S, Landon N, Liébault F, Dumont B, Richard-Mazet A (2009). Conceptual framework and interdisciplinary approach for the sustainable management of gravel-bed rivers: the case of the Drome River basin (S.E. France). Aquat Sci.

[CR51] Pont D, Logez M, Carrel G, Rogers C, Haidvogl G (2015) Historical change in fish species distribution: shifting reference conditions and global warming effects. Aquat Sci (this issue) 10.1007/s00027-014-0386-z PMC4525805 26257502

[CR52] Resh VH, Brown AV, Covich AP, Gurtz ME, Li HW, Minshall GW, Reice SR, Sheldon AL, Wallace JB, Wissmar RC (1988). The role of disturbance in stream ecology. J North Am Benthol Soc.

[CR53] Reyjol Y, Hugueny B, Pont D, Bianco PG, Beier U, Caiola N, Casals F, Cowx I, Economou A, Ferreira T, Haidvogl G, Noble R, de Sostoa A, Vigneron T, Virbickas T (2007). Patterns in species richness and endemism of European freshwater fish. Glob Ecol Biogeogr.

[CR54] Rhemtulla J, Mladenoff D (2007). Why history matters in landscape ecology. Landscape Ecol.

[CR55] Rick TC, Lockwood R (2013). Integrating paleobiology, archeology, and history to inform biological conservation. Conserv Biol.

[CR56] Rinne J, Hughes R, Calamusso B (2005). Historical changes in large river fish assemblages of the Americas.

[CR57] Segurado P, Branco P, Avelar AP, Ferreira MT (2015) Historical changes in the functional connectivity of rivers based on spatial network analysis and the past occurrences of diadromous species in Portugal. Aquat Sci (this issue)

[CR58] Sonnlechner C, Winiwarter V (1999) Recht und Verwaltung in grundherrschaftlichen Waldordnungen Niederösterreichs und Salzburgs (16.–18. Jahrhundert). In: Heyen EV (ed) Naturnutzung und Umweltschutz in der europäischen Rechts– und Verwaltungsgeschichte. Jahrbuch für europäische Verwaltungsgeschichte 11:57–85

[CR59] Steinberg T (1991). Nature incorporated: industrialization and the waters of New England.

[CR60] Steinmann P, Koch W, Scheuring L (1937). Die Wanderungen unserer Süßwasserfische. Zeitschrift für Fischerei und deren Hilfswissenschaften.

[CR61] Suding K, Higgs E, Palmer M, Callicott JB, Anderson CB, Baker M, Gutrich JJ, Hondula KL, LaFevor MC, Larson BMH, Randall A, Ruhl JB, Schwartz KZS (2015). Committing to ecological restoration. Science.

[CR62] Swetnam TW, Allen CD, Betancourt JL (1999). Applied historical ecology: using the past to manage the future. Ecol Appl.

[CR63] Syvitski JPM, Kettner A (2011). Sediment flux and the Anthropocene. Philos Transac Royal Soc.

[CR64] Szabo P (2014). Historical ecology: past, present and future. Biol Rev (online first).

[CR65] Szpak P (2011). Fish bone chemistry and ultrastructure: implications for taphonomy and stable isotope analysis. J Archaeol Sci.

[CR66] Tockner K, Uehlinger U, Robinson CT, Tonolla D, Siber R, Peter FD, Tockner K, Uehlinger U, Robinson CT (2009). Introduction to European rivers. Rivers of Europe.

[CR67] Torkar G, Zwitter Z (2015) Historical impacts of mercury mining and stocking of non-native fish on ichthyofauna in the Idrijca River Basin, Slovenia. Aquat Sci (this issue)

[CR68] Van Neer W (ed) (1994) Fish exploitation in the past. International Council for Archaeozoology, Fish Remains Working Group, Proceedings of the 7th meeting of the ICAZ Fish Remains Working Group. Annalen/Koninklijk Museum voor Midden-Afrika, Zoologische Wetenschappen 274, Tervuren, België

[CR69] Verheyen K, Honnay O, Bossuyt B, Hermy M, Honnay O, Verheyen K, Bossuyt B, Hermy M (2004). What history can teach us about present and future forest biodiversity. Forest biodiversity: Lessons from history for conservation.

[CR70] Walter RC, Merritts DJ (2008). Natural streams and the legacy of water-powered mills. Science.

[CR71] Ward J (1989). The four-dimensional nature of lotic ecosystems. J North Am Benthol Soc.

[CR72] Warde P (2006). Ecology, economy and state formation in early modern Germany.

[CR73] White PS, Pickett ST, Picket ST, White PS (1985). Natural disturbance and patch dynamics: an introduction. The ecology of natural disturbance and patch dynamics.

[CR74] Wiens JA, Hayward GD, Safford HD, Giffen C (2013). Historical environmental variation in conservation and natural resource management.

[CR75] Williams JW, Jackson ST (2007). Novel climates, no-analog communities, and ecological surprises. Front Ecol Environ.

[CR76] Winiwarter V (2004). Environmental history in Europe 1994–2004: enthusiasm and consolidation. Environ Hist.

[CR77] Wolter C (2015) Historic catches, abundance, and decline of Atlantic salmon Salmo salar in the River Elbe. Aquat Sci (this issue)

[CR78] Yurtseva A, Salmina E, Galik A, Lajus D (2015) How a millennium of fishing changed fish populations: a case study of Lake Peipus and the Velikaya River (NW Russia). Aquat Sci (this issue)

